# Retinal venous pressure in chronic smokers

**DOI:** 10.1186/s13167-015-0031-5

**Published:** 2015-04-08

**Authors:** Zakieh Vahedian, Heidar Amini, Mehdi Hosseini Tehrani, Reza Zarei, Sasan Moghimi, Maneli Mozaffarieh, Ghasem Fakhraie

**Affiliations:** Glaucoma Service, Farabi Eye Hospital, Tehran University of Medical Sciences, Qazvin Sq., South Kargar Ave., Tehran, 1336616351 Iran; Department of Ophthalmology, University of Basel, Basel, Switzerland

**Keywords:** Retinal venous pressure, Ophthalmodynamometric force, Smoking, Predictive preventive and personalized medicine

## Abstract

**Background:**

The overall aim of this study was to determine retinal venous pressure (RVP) in healthy chronic smokers and compare values to those of healthy non-smokers.

**Methods:**

Both eyes of 25 healthy chronic smokers and 41 healthy non-smokers were included. Measurements of RVP were performed by means of contact lens ophthalmodynamometry. Ophthalmodynamometry is done by applying increasing force on the eye via a contact lens. If a spontaneous venous pulsation was present, it was noted. If not, the compressive force was increased until the first venous pulsation was detected, and the measurement value was fixed and read. RVP was calculated as the sum of pressure increase induced by the instrument and intraocular pressure.

**Results:**

Smokers had a significantly higher frequency of spontaneous venous pulsations than non-smokers (*p* < 0.001). Mean values of RVP were slightly lower in smokers than in non-smokers: 15.3 and 15.5 (smokers) versus 15.9 and 16.2 (non-smokers) for the right and left eye, respectively; however, the difference in RVP between the two groups did not reach significance. There was no significant difference in blood pressure between the two groups, but heart rate was significantly higher in smokers (*p* = 0.043).

**Conclusions:**

RVP values may differ in healthy smokers than in non-smokers. Therefore, smoking habits should be considered when interpreting RVP results.

## Overview

Cigarette smoking is associated with an increased risk for vascular disease [1-3]. The positive relationship between smoking and coronary heart disease deaths in men was first reported in 1940 [4], with the risk up until today being strongly dose related [5]. Smoking nearly doubles the risk of ischemic stroke [6] and more than doubles the number of DNA breaks at a time in the healthy persons’ circulating leukocytes [7]. Smokers are at risk for a variety of chronic vascular diseases including peripheral arterial disease, stroke, heart attack, abdominal aortic aneurysm and subsequent death [8,9]. The acute cardiovascular effects of tobacco smoking have been attributed mainly to nicotine. Nicotine releases catecholamines via stimulation of the sympathetic ganglia [10,11].

Smoking elicits an increase in sympathoadrenergic tone resulting in increase in the heart rate, blood pressure and blood levels of catecholamines such as adrenaline [8,12,13]. The effect of acute exposure of nicotine on vascular resistance, however, is somewhat controversial [14-16]. Some evidence suggests that vascular resistance in chronic smokers may even be lower than that in non-smokers [17].

Little is known about the hemodynamic effects of nicotine on ocular circulation. In 1993, Rojanapongpun and Drance found that small doses of nicotine (Nicorette gum) increased blood flow velocities in the ophthalmic artery of glaucoma patients whereas it significantly decreased finger blood flow [18]. The authors, however, did not state whether the participants were smokers or not. In 1994, Morgado et al. [19] found a decreased retinal blood flow in acute smokers, and 1 year later, Williamson et al. [20] found that cigarette smoking was associated with lower ophthalmic artery velocities. Some years later in 1997, Kaiser and Flammer observed that in the majority of the measured ocular vessels, blood flow velocity was higher in smokers than in non-smokers [17]. Recently, Kalpana and colleagues observed that in smokers, normoxic hypercapnia resulted in a significant increase in velocity flow and total venous retinal blood flow [21].

Retinal venous pressure (RVP) is an important parameter in the clinical assessment of retinal blood flow. It is assumed that in healthy people, RVP commonly equals the intraocular pressure (IOP); in other words, healthy people commonly have a spontaneous venous pulsation [22-26]. This assumption, however, is not always correct for healthy people [22,25-27] neither for those with a disease (e.g. glaucoma) [27-33].

Taking the above information into account, we wondered whether RVP in healthy chronic smokers would be any different to healthy non-smokers. The overall aim of this study was to measure RVP in healthy chronic smokers (during a smoking-free period) and compare values to healthy non-smokers.

## Methods

In this cross sectional case-control study, participants were invited by an ophthalmologist (ZV) of the Farabi Eye Hospital in Tehran to participate in this study on a volunteer basis and receive a routine ophthalmological checkup in return. The study was approved by the local ethical committee of Tehran University of Medical Sciences, and all participants gave their consent to participate in the study. Included were 25 smokers and 41 people who did not smoke at all. Excluded were subjects under local or systemic medication as well as subjects with a history of eye or general disease. None of the participants had any pathological findings (other than increased retinal venous pressure) in their routine ophthalmological examination. The non-contact examination was a part of our routine ophthalmoscopic examination of the fundus of the patients. Blood pressure examination and heart rate were measured in all participants. RVP was measured in both eyes of all participants by means of an ophthalmodynamometer. All measurements were performed by the same ophthalmologist (ZV). The interval between the last cigarette smoked and the time of RVP measurement was 30 to 60 min.

RVP was measured in both eyes by ophthalmodynamometry (IMEDOS, Jena, Germany) [34]. This device consists of a conventional Goldmann contact lens fitted with a pressure sensor at its outer margin where the Goldmann contact lens is usually held during an ophthalmoscopic examination. The contact lens is fitted to an outer ring by strain gauges which give an electrical signal. This signal is linearly related to the force by which the contact lens is attached to the eye. It is given to the input of a central unit by a thin flexible cable. This central unit has the size of a pocket calculator. It shows the increase of the IOP induced by the force applied on a LCD display. The conversion from force to pressure is based on a biophysical calibration [35,36].

Ophthalmodynamometry was conducted by applying increasing force to the eye via the contact lens. This applied pressure can be read as a pressure increase on the attached LCD screen based on a calibration curve. Any small pulsatile synchronous movement of the central retinal vein or its major branches inside the optic disc was noted as spontaneous pulsation. In short, after placing the CLD on the eye, the ONH was brought into sight. If a spontaneous venous pulsation was present, it was noted. If not, the compressive force was increased until the first venous pulsation was detected, and the measurement value was fixed and read. RVP was calculated as the sum of pressure increase induced by the instrument and IOP.

### Statistical analysis

Statistical analysis was performed using SPSS 16.0 software (SPSS Inc, Chicago, Illinois, USA). Because of the small sample size, non-parametric tests were used to analyze the data. Comparison of quantitative variables between smokers and non-smokers was performed using Mann-Whitney *U* test. The difference in categorical data was evaluated using chi-square test. A *p* value <0.05 was considered statistically significant.

## Results

The patient characteristics are listed in Table 1. The mean number of cigarettes smoked per day by smokers was 25 (SD 8.84) with a range of 10 to 40.

**Table 1:** **Patient characteristics of the two groups**

**Variable**	**Smokers**	**Non-smokers**	***p*** **value**
Number	25	41	
Age, mean (SD)^a^	44.16 (10.65)	42.7 (13.1)	n.s.
Gender (M/F)	18/7	20/21	n.s.
Systolic BP (mmHg), mean (SD)^a^	123.2 (21.0)	120.6 (28.7)	n.s.
Diastolic BP (mmHg), mean (SD)^a^	71.2 (11.9)	68.9 (14.1)	n.s.
Heart rate (beat/min), mean (SD)^a^	77.5 (7.3)	74.0 (9.3)	0.043^a^
IOP right eye (mmHg), mean (SD)^a^	14.2 (2.9)	13.0 (2.4)	n.s.
IOP left eye (mmHg), mean (SD)^a^	14.6 (3.1)	13.1 (2.4)	n.s.

*M* male, *F* female, *BP* blood pressure, *IOP* intraocular pressure, n.s. = not significant.

^a^Independent Mann-Whitney *U* test.

**Figure 1:**
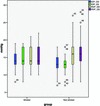
**Box plot of intraocular pressure (IOP) and retinal venous pressure (RVP) in both eyes of smokers and non-smokers.** OD = right eye, OS = left eye.

Smokers had a significantly higher frequency of spontaneous venous pulsations than non-smokers (86.0% vs 51%, *p* < 0.001). Mean values of RVP were slightly lower in smokers than in non-smokers: 15.3 and 15.5 (smokers) versus 15.9 and 16.2 (non-smokers) for the right and left eye, respectively; the difference in RVP, however, between the two groups did not reach significance (Figure 1). There was no significant difference in blood pressure between the two groups, but heart rate was significantly higher in smokers (*p* = 0.043, Table 1).

No significant differences were accounted for in the IOP values between the smoking and non-smoking group or between the two eyes of patients within each group (Table 1).

### Discussion

In our study, smokers had a slightly lower mean RVP and a higher frequency of spontaneous retinal vein pulsations in comparison to age- and sex-matched healthy non-smokers. Moreover, 49% of the non-smoking healthy participants lacked a spontaneous venous pulsation, a value which is higher than those of previous reports. How can we explain these observations?

Reports of spontaneous venous pulsations in healthy subjects vary. Whereas some reports state that only approximately 2% of the healthy population does not have spontaneous venous pulsations [25], others report the lack of a spontaneous venous pulsation in approximately 10%–24% of healthy subjects [22,25-27,37]. Our results of 49% suggest that these values may possibly vary from population to population, with an increased RVP being more frequent in healthy people than previously accounted for.

Findings of a higher frequency of spontaneous venous pulsation in smokers go hand in hand with results of previous studies showing a significantly higher blood flow velocity in the majority of the ocular vessels of smokers. Back in 1997, Kaiser and Flammer [17] discussed that the smoke of a cigarette besides many other components contains approximately 2%–6% carbon monoxide [38] leading to an increase in carboxyhemoglobin levels up to 3%–15% in smokers compared to a value of less than 1.5% in non-smokers [39]. The relative oxygen deficit would provoke regulatory effects leading to downstream vasodilation and an increase in blood flow velocity. Our results support this argument; not only was the frequency of spontaneous venous pulsations higher in smokers but also mean values of RVP were slightly lower in smokers compared to non-smokers. Nevertheless, it should be noted that differences in the methodology amongst various institutions such as the method of ophthalmoscopy or the pulsation criterion set by the examiner to define as spontaneous venous pulsation may lead to different results. Our definition of a spontaneous venous pulsation was the same as that of our colleagues Stodtmeister et al. [33].

There was no difference in systolic or diastolic blood pressure in the two groups. These data are somewhat paradoxical because cigarette smoking elicits an increase in sympathoadrenergic tone, resulting in the elevation of blood pressure [40,41]. We can only hypothesize what is happening, yet this discrepancy may be explained as follows. Initially, a vasoconstriction mediated by nicotine causes an increase in systolic blood pressure [42]. This phase is followed by a decrease in blood pressure as a consequence of depressant effects played chronically by nicotine itself [43]. Moreover, healthy cigarette smokers have an increased sensitivity to endogenous endothelium-dependent and endothelium-independent vasodilators [44]. Assuming that the increase in adrenergic tone caused by nicotine is counteracted to some degree by the increased sensitivity to endogenous vasodilator stimuli, the equalization in blood pressure between the two groups may be explained.

This study has some limitations that should be kept in mind; the sample size is small with specific reference to the smoking group. More studies with larger sample sizes can help us infer more reliable assumptions about our population. Moreover, differences in RVP between the two groups may have reached significance had the sample size been large enough.

### Conclusion

Altogether, RVP values appear to be different in healthy smokers than in non-smokers. Future study designs of RVP measurements should, therefore, take into account the differences between smokers and non-smokers.

## Abbreviations

RVP: Retinal venous pressure
IOP: Intraocular pressure
BP: Blood pressure
